# Überversorgung in der Intensivmedizin: erkennen, benennen, vermeiden: Positionspapier der Sektion Ethik der DIVI und der Sektion Ethik der DGIIN

**DOI:** 10.1007/s00063-021-00794-4

**Published:** 2021-03-01

**Authors:** Andrej Michalsen, Gerald Neitzke, Jochen Dutzmann, Annette Rogge, Anna-Henrikje Seidlein, Susanne Jöbges, Hilmar Burchardi, Christiane Hartog, Friedemann Nauck, Fred Salomon, Gunnar Duttge, Guido Michels, Kathrin Knochel, Stefan Meier, Peter Gretenkort, Uwe Janssens

**Affiliations:** 1 Klinik für Anästhesiologie, Intensivmedizin, Notfallmedizin und Schmerztherapie, Klinikum Konstanz, Konstanz, Deutschland; 2 Institut für Geschichte, Ethik und Philosophie der Medizin, Medizinische Hochschule Hannover, Hannover, Deutschland; 3 Universitätsklinik und Poliklinik für Innere Medizin III, Universitätsklinikum Halle (Saale), Halle (Saale), Deutschland; 4 Geschäftsbereichs der Medizinethik, Christian-Albrechts-Universität zu Kiel, Kiel, Deutschland; 5 Institut für Ethik und Geschichte der Medizin, Universitätsmedizin Greifswald, Greifswald, Deutschland; 6 Institut für Biomedizinische Ethik und Geschichte der Medizin, Universität Zürich, Zürich, Schweiz; 7 Bovenden, Deutschland; 8 Klinik für Anästhesiologie und Intensivmedizin, Charité Universitätsmedizin Berlin, Berlin, Deutschland; 9 Patienten- und Angehörigenzentrierte Versorgung (PAV), Klinik Bavaria, Kreischa, Deutschland; 10 Klinik für Palliativmedizin, Georg-August-Universität Göttingen, Göttingen, Deutschland; 11 Lemgo, Deutschland; 12 Abteilung für strafrechtliches Medizin- und Biorecht, Georg-August-Universität Göttingen, Göttingen, Deutschland; 13 Klinik für Akut- und Notfallmedizin, St.-Antonius-Hospital Eschweiler, Eschweiler, Deutschland; 14 Kinderklinik und Kinderpoliklinik im Dr. von Haunerschen Kinderspital Kinderpalliativzentrum, Klinikum der Universität München, München, Deutschland; 15 Ethik der Medizin und Gesundheitstechnologie, Technische Universität München, München, Deutschland; 16 Klinik für Anästhesiologie, Universitätsklinikum Düsseldorf, Düsseldorf, Deutschland; 17 Simulations- und Notfallakademie, Helios Klinikum Krefeld, Krefeld, Deutschland; 18 Klinik für Innere Medizin und Internistische Intensivmedizin, St.-Antonius-Hospital Eschweiler, Dechant-Deckers-Str. 8, 52249 Eschweiler, Deutschland

**Keywords:** Therapie am Lebensende, Behandlungsausmaß, Patientenversorgung, Therapieziel, Ärztliche Indikation, Patientenwille, End of life care, Patient care, Extent of treatment, Therapeutic goal, Patient preference

## Abstract

Ungeachtet der sozialgesetzlichen Vorgaben existieren im deutschen Gesundheitssystem in der Patientenversorgung nebeneinander Unter‑, Fehl- und Überversorgung. Überversorgung bezeichnet diagnostische und therapeutische Maßnahmen, die nicht angemessen sind, da sie die Lebensdauer oder Lebensqualität der Patienten nicht verbessern, mehr Schaden als Nutzen verursachen und/oder von den Patienten nicht gewollt werden. Daraus können hohe Belastungen für die Patienten, deren Familien, die Behandlungsteams und die Gesellschaft resultieren. Dieses Positionspapier erläutert Ursachen von Überversorgung in der Intensivmedizin und gibt differenzierte Empfehlungen zu ihrer Erkennung und Vermeidung. Zur Erkennung und Vermeidung von Überversorgung in der Intensivmedizin erfordert es Maßnahmen auf der Mikro‑, Meso- und Makroebene, insbesondere die folgenden: 1) regelmäßige Evaluierung des Therapieziels im Behandlungsteam unter Berücksichtigung des Patientenwillens und unter Begleitung von Patienten und Angehörigen; 2) Förderung einer patientenzentrierten Unternehmenskultur im Krankenhaus mit Vorrang einer qualitativ hochwertigen Patientenversorgung; 3) Minimierung von Fehlanreizen im Krankenhausfinanzierungssystem gestützt auf die notwendige Reformierung des fallpauschalbasierten Vergütungssystems; 4) Stärkung der interdisziplinären/interprofessionellen Zusammenarbeit in Aus‑, Fort- und Weiterbildung; 5) Initiierung und Begleitung eines gesellschaftlichen Diskurses zur Überversorgung.

## Abstract (en)

Despite social laws, overtreatment, undertreatment, and incorrect treatment are all present in the German health care system. Overtreatment denotes diagnostic and therapeutic measures that are not appropriate because they do not improve the patients’ length or quality of life, cause more harm than benefit, and/or are not consented to by the patient. Overtreatment can result in considerable burden for patients, their families, the treating teams, and society. This position paper describes causes of overtreatment in intensive care medicine and makes specific recommendations to identify and prevent it. Recognition and avoidance of overtreatment in intensive care medicine requires measures on the micro-, meso- and macrolevels, especially the following: (1) frequent (re-)evaluation of the therapeutic goal within the treating team while taking the patient’s will into consideration, while simultaneously attending to the patients and their families; (2) fostering a patient-centered corporate culture in the hospital, giving priority to high-quality patient care; (3) minimizing improper incentives in health care financing, supported by reform of the reimbursement system that is still based on diagnose-related groups; (4) strengthening of interprofessional co-operation via education and training; and (5) initiating and advancing a societal discourse on overtreatment.

## Einleitung

Nach Einschätzung der Organisation für Entwicklung und Zusammenarbeit in Europa die Organisation for Economic Co-operation and Development (OECD) wird etwa ein Fünftel der Gesundheitsausgaben in den Mitgliedsländern für Leistungen verwendet, die keinen oder nur einen marginalen Beitrag für bessere Gesundheitsergebnisse leisten [56]. Ungeachtet der sozialgesetzlichen Vorgaben (§§ 2 Abs. 1 und 4,12 Sozialgesetzbuch [SGB] V) existieren im deutschen Gesundheitssystem in der Patientenversorgung nebeneinander Unter‑, Fehl- und Überversorgung [20, 33, 65].

Das vorliegende Positionspapier richtet den Blick auf die Überversorgung in der Intensivmedizin [14, 31, 62]. Ziel der Intensivmedizin ist es, lebensbedrohliche medizinische Krisen zu überbrücken, damit die Patientinnen nach der intensivmedizinischen Behandlung weitgehend selbstbestimmt und mit einer für sie akzeptablen Lebensqualität außerhalb der Intensivstation weiterleben können. Grundsätzlich müssen für jede (intensiv)medizinische Behandlung erstens eine Indikation und zweitens eine rechtswirksame Einwilligung durch die Patientin oder ihren Stellvertreter vorliegen (sog. Zwei-Säulen-Modell; [17]). Die Behandlungsteams müssen also – auch wiederholt – die jeweilige Prognose bewerten und abwägen, welche der häufig komplexen Behandlungsmaßnahmen medizinisch sinnvoll für die individuelle Patientin unter Berücksichtigung ihres Willens und ihrer Wertvorstellungen angewandt werden können und dürfen. Es stellt somit eine große Herausforderung und Verantwortung dar, den jeweils angemessenen Behandlungsumfang zu bestimmen.

Überversorgung bezeichnet Behandlungsmaßnahmen, die *nicht* angemessen sind, weil sie zu keiner für die Patientin bedeutsamen Verbesserung der (Über‑)Lebensdauer oder Lebensqualität führen, mehr Schaden als Nutzen verursachen und/oder von Patientinnen nicht gewollt werden [51, 56]. Überversorgung ist also nicht von Bedarf und Bedürfnis der Patientin in ihrer individuellen Krankheitssituation, sondern durch andere Motive veranlasst. Überversorgung kann mit hohen Belastungen und Risiken für die betroffenen Patientinnen, ihre Familien und die Behandlungsteams verbunden sein; sie kann Leiden und Trauer verursachen oder verlängern sowie zu Gewissensnot, „moral distress“, Burn-out und Personalabwanderung beitragen [40, 44]. Überversorgung schadet der Allgemeinheit, weil die dadurch gebundenen Ressourcen für andere Zwecke nicht mehr zur Verfügung stehen (Opportunitätskosten). Außerdem kann eine Überversorgung in der Intensivmedizin in allen nachfolgenden Bereichen des Gesundheitssystems eine ungerechtfertigte Versorgungskaskade verursachen. Insofern ist es dringlich geboten, Überversorgung auch in der Intensivmedizin zu erkennen und zu vermeiden. Darüber hinaus ist es wichtig zu verdeutlichen, dass Therapielimitierungen nicht das Ende aller medizinischen und pflegerischen Behandlung bedeuten. Angebote der palliativmedizinischen Behandlung und Begleitung sind darauf ausgerichtet, körperliches und psychosoziales Leid zu lindern und Patientinnen und ihre An- und Zugehörigen in der letzten Lebensphase nicht allein zu lassen.

## Ursachen für Überversorgung

**Figure 1:**
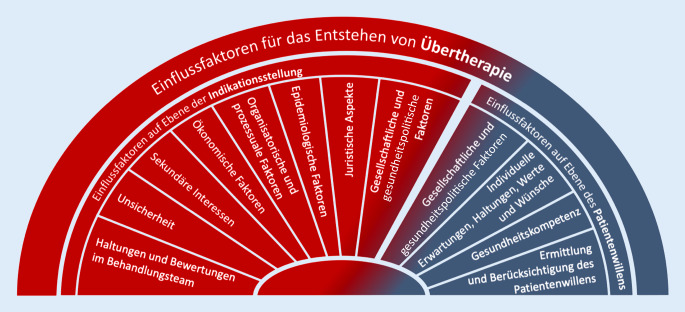

Gesellschaftliche Wertvorstellungen und Übereinkünfte, Organisations- und Versorgungsstrukturen des Gesundheitswesens, demographische und medizintechnische Entwicklungen, rechtliche Regelungen, kommerzielle Interessen und insbesondere die individuelle Arzt-Patienten-Beziehung beeinflussen mit unterschiedlicher Wirkkraft den Behandlungsumfang. Diese unterschiedlichen Faktoren können damit auch Überversorgung begünstigen oder vermeiden. Während die beiden Säulen „Indikation“ und „Patientenwille“ für die Behandlungsentscheidung gleichermaßen relevant sind, beeinflussen sie Überversorgung in unterschiedlicher Weise (Abb. 1).

### Einflussfaktoren bei der Indikationsstellung

#### Haltungen und Bewertungen innerhalb des Behandlungsteams

Indikationsstellungen werden im Alltag nicht nur von objektiven Fakten, sondern auch von persönlichen, intuitiven und emotionalen Bewertungen zur Sinnhaftigkeit der jeweiligen medizinischen Maßnahmen bestimmt [48]. Dazu gehören auch die etabliert vermittelten und erlernten Fähigkeiten zur kritischen Reflexion, die Prägung durch die Fachzugehörigkeit und das berufliche Umfeld, das Menschenbild, die Religiosität und andere soziokulturelle Faktoren [17, 74].

Vielfach stellen persönliche Faktoren der Behandelnden substanzielle Ursachen für den Versorgungsumfang dar [34, 65]. Beispielsweise wird die grundsätzlich positive Einstellung, Leben retten und erhalten zu wollen, dann zu einer negativen und gefährlichen Haltung, wenn die Rettung auch in erkennbar aussichtslosen Fällen versucht wird. Selbstüberschätzung und unbewusste Allmachtsphantasien können dabei eine Rolle spielen. Es fällt oft leichter, alle zur Verfügung stehenden Mittel unreflektiert anzuwenden, als differenziert und individuell nach Therapiezielen und Erfolgsaussicht zu fragen („Retten um jeden Preis“, „rule of rescue“ [29]). Die Tabuisierung von Sterben und Tod auch innerhalb der Behandlungsteams und das Erleben von Tod als persönliches Versagen können diesen Effekt verstärken. Eine „schöngeredete“ Einschätzung der Prognose führt in diesen Fällen zu letztlich sinnlosen Therapiebemühungen.

Forschungsergebnisse aus der Psychologie der Entscheidungsfindung benennen unwillkürliche Entscheidungsfehler, die zur Übertherapie führen können [58], beispielsweise:

„Sunk cost effect“: Behandler halten an einer einmal getroffenen Entscheidung fest, weil sie bereits viel investiert haben und diese Investition nicht „umsonst“ gewesen sein soll. Dieser Effekt kann sich so weit verstärken, dass immer mehr und immer sinnlosere Aktionen unternommen werden.

„Omission bias“ („Unterlassungseffekt“): Stirbt eine Patientin, nachdem das Behandlungsteam entschieden hat, den Behandlungsumfang zu limitieren, erscheint dies subjektiv schlimmer, als dass die Patientin unter einer fortlaufenden Therapie verstirbt.

#### Unsicherheit

Übertherapie kann auch durch Unsicherheit verursacht sein, einerseits durch mangelnde Kenntnis des aktuellen medizinischen Wissensstands sowie der ethischen und juristischen Rahmenbedingungen, insbesondere hinsichtlich der „end-of-life care“ [17, 65]. Andererseits ist Medizin bei der Einschätzung der Therapiewirksamkeit oder hinsichtlich prognostischer Aussagen für den individuellen Einzelfall immer von Unsicherheiten geprägt [16]. Diese Unsicherheit muss vom Behandlungsteam für den Einzelfall wahrgenommen, eingeordnet, bewertet und in Entscheidungsprozesse einbezogen werden, um Fehlversorgung generell zu reduzieren [81]. Unsicherheiten müssen insbesondere im Arzt-Patienten-Gespräch ehrlich und empathisch angesprochen werden.

#### Konflikte bei sekundären Interessen

Das primäre ärztliche Interesse muss grundsätzlich die bestmögliche Behandlung jeder Patientin sein [28]. Auf der individuellen Ebene können Konflikte bei sekundären Interessen, wie die Gewährung oder Annahme finanzieller Vorteile (z. B. Bonus- oder Beraterverträge) oder die Verbesserung des sozialen Status (z. B. Wunsch nach Anerkennung oder Karrierestreben), das Risiko einer Überversorgung erhöhen [5, 38].

#### Überbetonung ökonomischer Faktoren

Die Einführung des deutschen Fallpauschalensystems im Jahr 2002 (basiert auf „diagnosis-related groups“, DRG) hat den ökonomischen Druck auf die Krankenhäuser politisch intendiert verschärft [12]. Dabei wurde versäumt, die Einführung von Fallpauschalen im Krankenhauswesen mit Strukturveränderungen zu verbinden [61, 72]. Die Ökonomisierung deutscher Krankenhäuser hat dazu geführt, dass Erlösoptimierung zu einem wesentlichen Unternehmensziel geworden ist. Dieser ökonomische Druck beeinflusst die Intensität der Leistungen und damit implizit auch die Behandlungsentscheidungen. Bei privaten Krankenhausträgern ist besonders problematisch, dass dort Gewinne erwirtschaftet werden sollen, die zumindest potenziell dem Gesundheitssystem entzogen und zweckentfremdet Aktionären zugeführt oder in gesundheitsfernen Bereichen investiert werden können [75]. Die Bundesländer hingegen kommen ihrer Verpflichtung zur Übernahme der Investitionsmittel für die Krankenhäuser nur unzureichend nach. Dies schafft Einfallstore für Überversorgung durch Erzwingen eines betriebswirtschaftlich begründeten Verhaltens, führt in der Fläche zu ineffizienten Versorgungsstrukturen und provoziert eine unzulässige Quersubventionierung aus Beitragsmitteln der gesetzlichen Krankenversicherungen in Milliardenhöhe.

Das System der DRG gibt den Kliniken Anreize zu Mengensteigerungen, insbesondere bei Maßnahmen, bei deren Vergütung rechnerisch ein hoher Anteil auf Fixkosten entfällt. Operative und interventionelle Leistungen werden dabei häufig besser vergütet als konservative Behandlungsmaßnahmen oder ein ausführliches Arzt-Patienten-/-Angehörigen-Gespräch. Dies erhöht das Risiko einer Überversorgung. In den letzten Jahren stieg beispielsweise die Inanspruchnahme von Intensivtherapie jährlich mit 3 % deutlich stärker als die Inanspruchnahme von Krankenhausbehandlung insgesamt (0,8 %), vor allem am Lebensende. Inzwischen erhält jede/r vierte aller Patienten, die in einem Krankenhaus versterben, eine Intensivtherapie [18].

Die per se kostenintensiven Intensivstationen sind inzwischen zentral in das System der wirtschaftlich orientierten Krankenhausunternehmen eingebettet. Insbesondere die Kodierungsmöglichkeit „intensivmedizinische Komplexbehandlung“ (OPS 8.98 f.) verleitet zur exzessiven Nutzung, also Gewinnabschöpfung, ohne dass der entsprechende Behandlungsumfang tatsächlich immer erforderlich ist. Hingegen spielen Maßnahmen zur Verbesserung einer patientenorientierten Indikations- und Ergebnisqualität im aktuellen Finanzierungssystem keine regulierende Rolle [61].

#### Organisatorische und prozessuale Faktoren

Die Gewinnoptimierung wird wesentlich durch Einsparungen im Personalbereich bei gleichzeitiger Ausweitung von Leistungen erzielt. Eine Mehrbelastung des Behandlungsteams durch eine (geplante) personelle ärztliche und/oder pflegerische Unter- oder Fehlbesetzung hat erhebliche negative Auswirkungen auf die Gesundheit des Personals und die Gesundung der Patientinnen [40, 44, 52, 76]. Personalunterdeckung erfordert ein (subjektiv) selektives Auswählen von Teilaufgaben – also ggf. auch eine implizite Rationierung – und schränkt die Möglichkeit ein, das Behandlungsziel interprofessionell und interdisziplinär zu reflektieren. Dadurch wird ein unkritisches Einleiten oder Fortsetzen von Therapiemaßnahmen befördert, was einer Überversorgung Vorschub leistet [80, 82].

An den Schnittstellen Übergabe und Visite besteht durch mangelhaft strukturierte Durchführung und Dokumentation das Risiko von Informationsverlusten [3, 37]. Nachfolgend kann es dann beispielsweise zu erneuten, nicht notwendigen oder auch patientenseits nicht gebilligten diagnostischen oder therapeutischen Maßnahmen kommen. Dies führt nicht selten einerseits zu Rückschritten im Genesungsprozess und andererseits zu längeren Krankenhausverweildauern [3] und steigert die Opportunitätskosten. Insbesondere wenn Entscheidungen zur Therapielimitierung nicht rechtzeitig getroffen und kommuniziert werden, erfolgt häufig eine sinnlose Therapieeskalation.

Das „ethische Klima“ einer Intensivstation fasst die gemeinsame Denk- und Handlungsweise des Behandlungsteams zusammen. Damit beeinflusst das ethische Klima auf einer Intensivstation wesentlich und wirksam, ob

- Therapieziele frühzeitig angesprochen und deren Erreichbarkeit regelmäßig überprüft werden,
- die (Tages‑)Therapieziele patientenrelevant sind oder lediglich der Korrektur (patho)physiologischer Parameter dienen,
- Entscheidungen zu Therapiekonzept und -umfang im Team getroffen werden,
- Therapiebegrenzungen zugelassen oder vermieden werden und
- eine patienten- und angehörigenzentrierte Versorgung stattfindet.

Das ethische Klima wird entscheidend durch Haltung und Handeln der ärztlichen und pflegerischen Leitungspersonen sowie das Miteinander der verschiedenen beteiligten Fachdisziplinen bestimmt. Ein mangelhaftes ethisches Klima begünstigt Überversorgung, ein gutes ethisches Klima hingegen begünstigt einen berufsgruppen- und fachübergreifenden Diskurs und einen individuell angemessenen Behandlungsumfang [4, 41, 76].

#### Juristische Faktoren

Die Sorge vor juristischen, d. h. haftungs- oder gar strafrechtlichen Konsequenzen ist seit langem als bedeutsamer Faktor für Überdiagnostik und Übertherapie bekannt. Um nicht wegen unterlassener Hilfeleistung oder eines Behandlungsfehlers belangt zu werden, scheuen nicht nur weniger erfahrene Behandelnde vor einer Verantwortungsübernahme für therapiebegrenzende Entscheidungen zurück („juristische Indikation“; [78]) und warten zögerlich lieber auf einen „Wink des Schicksals“. Dabei birgt die Hintanstellung des Patientenwohls selbst rechtliche Risiken, von den professionsethischen Bedenken gegenüber einer solchen „Defensivmedizin“ ganz abgesehen.

Eine wesentliche Ursache hierfür ist der Umstand, dass einschlägige gesetzliche Vorschriften, juristische Begriffe und Gerichtsentscheidungen dem Behandlungsteam unzureichend bekannt sind oder nicht zutreffend interpretiert werden. Auf die Einordnung juristischer Aspekte sind (angehende) Ärztinnen bislang jedenfalls nicht ausreichend vorbereitet. Auch das Rechtssystem achtet zu wenig auf das Ziel der Orientierungssicherheit. Widerstreitende Rechtsmeinungen, mitunter gegenläufige Urteile sowie ein Hang zur Überdifferenzierung erzeugen elementare Unsicherheiten der Behandelnden bei Therapieentscheidungen. Das wiegt umso schwerer, als die gesellschaftliche Akzeptanz der Rechtsprechung rechtssoziologisch nicht ernsthaft evaluiert oder hinterfragt wird. Erforderliche Entscheidungen zur Therapiebegrenzung werden vor diesem Hintergrund nicht getroffen oder hinausgezögert [15].

#### Gesellschaftliche und politische Faktoren

In Deutschland muss bislang niemand auf die zügige und gründliche Behandlung einer schweren Erkrankung verzichten. Die ständigen technologischen und pharmakologischen Weiterentwicklungen in der Medizin, das Interesse verschiedener Institutionen, an einem wirtschaftlich attraktiven Wachstumsmarkt teilzuhaben, und die demographische Entwicklung leisten aber einer künftigen Mittelknappheit in der medizinischen Versorgung Vorschub. Des ungeachtet findet in der Öffentlichkeit bisher eine offene und vor allem transparente Diskussion über die Grenzen des medizinischen Versorgungsystems in diesem Spannungsfeld und über die Überversorgung *nicht* statt. Vielmehr wurde die Erwartungshaltung der Bevölkerung an eine „grenzenlose Medizin“ durch ein zunehmend gewinnorientiertes und von privaten Trägern gesteuertes Gesundheitssystem weiter befeuert.

Die Maßlosigkeit sowohl vieler Nutzer des medizinischen Versorgungssystems als auch derjenigen, die bei ihnen ungerechtfertigte Begehrlichkeiten wecken, verstärken und befriedigen, lässt allzu oft vergessen, dass Gesundheit prinzipiell ein öffentliches Gut darstellt. Die Bewahrung von Gesundheit muss solidarisch getragen, finanziert und durch ein grundsätzliches ärztliches Versprechen zur Hilfeleistung aufrechterhalten werden können [36, 45]. Überversorgung trägt maßgeblich dazu bei, die Aufrechterhaltung einer solidarischen Gesundheitsversorgung, an der alle Patienten teilhaben können, langfristig zu gefährden.

#### Epidemiologische Faktoren

Gesellschaftliche Vorstellungen von Gesundheit und Krankheit, Erwartungen an die Leistungsfähigkeit der Medizin und bestehende Krankheitsdefinitionen beeinflussen sich wechselseitig. Beispielsweise fehlt gerade auch der Intensivmedizin bis heute ein altersadaptiertes Gesundheitskonzept. Jedenfalls müssen Krankheitsdefinitionen, therapierelevante Grenzwerte und diagnostische oder therapeutische Vorgehensweisen regelmäßig auf ihre Evidenz überprüft und ggf. angepasst werden. Änderungen können allerdings auch ohne Vorliegen einer ausreichenden Evidenz oder aufgrund illegitimer Interessen herbeigeführt werden. Das kann zu einer scheinbaren Zunahme von Inzidenz und Prävalenz sowie einer Überschätzung der Effektivität und Unterschätzung von Nebenwirkungen führen. Daraus lässt sich eine vermeintliche Behandlungsnotwendigkeit konstruieren. Eine dann resultierende Übertherapie nutzt ökonomischen und/oder sekundären Interessen und bewirkt jedenfalls einen ungerechtfertigten Ressourcenverbrauch und unter Umständen auch Schädigungen der Patientinnen [6, 7, 25, 26, 59].

### Einflussfaktoren auf den Patientenwillen

#### Formung des Patientenwillens

Die Bedeutung von Gesundheit und Krankheit und die damit verbundenen Forderungen nach Behandlungsmaßnahmen sind tief verwurzelt in den Werten von Familie, von soziokultureller Prägung und Einbindung sowie von Bildungsgrad, Religion und Religiosität [27, 39, 73, 74]. Denkmuster, Verhaltensweisen und Erwartungshaltungen der Patientinnen an die moderne Medizin sowie das ständig wachsende Angebot des Gesundheitssystems beeinflussen – wissentlich oder auch unbewusst – eine Überdiagnostik und -therapie. Dazu gehören z. B. das fehlende Verständnis für eine (Rest‑)Ungewissheit, die der Medizin inhärent ist, ebenso wie die Inakzeptanz von „Abwarten“ und „Beobachten“ als Strategien in der Diagnosestellung und Therapieentwicklung [21].

Manche Patientinnen empfinden die eigene potenzielle Überversorgung insgesamt eher als eine besonders gute, fürsorgliche und großzügige Sorge um ihre Gesundheit und fordern diese ein [21]. Trotz einer Ahnung, dass Überversorgung existiert, fürchten sie für die eigene Behandlung eher Defizite und Restriktionen [21]. Möglicherweise tendieren Angehörige und gesetzliche Stellvertreter, besonders wenn letzteren die Persönlichkeit der Patientin und der familiäre Hintergrund noch nicht ausreichend bekannt sind, eher zur Übertherapie, weil sie im Licht ihrer Pflicht zum „Wohltun“ den Vorwurf einer „Untertherapie“ befürchten [47, 49].

Die lebensbedrohliche Situation während des intensivmedizinischen Aufenthalts kann die Patientin und ihr Umfeld in eine emotionale Ausnahmesituation versetzen und die Entscheidungsfähigkeit beeinflussen. So kann eine Anspruchshaltung auf eine intensivmedizinische Maximaltherapie nicht nur auf fehlendem Wissen und/oder einer Fehleinschätzung der Möglichkeiten und Risiken moderner Intensivmedizin, sondern auch auf Überforderung und kognitiver Abstumpfung basieren.

Zusätzlich führen auch Kommunikationsdefizite und Schwierigkeiten bei der Interpretation übermittelter Informationen zu Unsicherheiten und der Forderung nach (sog.) Maximaltherapie. Zwar steht inzwischen durch den informationstechnologischen Fortschritt allen (potenziellen) Patientinnen und Angehörigen eine Fülle an Informationen zur Verfügung; oft verhindern jedoch deren fragliche Qualität und die mangelnde Gesundheitskompetenz eine individuell angemessene Einschätzung [42, 53]. In der Informationsflut bleibt insbesondere häufig unbeachtet, dass die eingeforderte Maximaltherapie zum Überleben mit schwersten Einschränkungen führen kann [66].

#### Ermittlung und Berücksichtigung des Patientenwillens

Ärztliche Aufgabe ist es, die Indikation und den Patientenwillen zu einer Behandlungsentscheidung zusammenzuführen (Patientenrechtegesetz; § 1901a BGB; [17, 48]). Schon die Eruierung bzw. Interpretation des Patientenwillens auf Intensivstationen scheitert häufig an vielfältigen Problemen wie Akuität der Erkrankung, fehlende Einwilligungsfähigkeit der Patienten oder Unklarheiten über die (gesetzliche) Stellvertreterregelung und die Anwendbarkeit von Vorausverfügungen im konkreten Fall [19, 46, 47, 64]. Gespräche mit Patientinnen und Angehörigen bzw. gesetzlichen Stellvertretern sollen daher einerseits zu deren angemessener Information und Aufklärung dienen. Andererseits sollen diese Gespräche auch dazu beitragen, dass das Behandlungsteam die Legitimation diagnostischer und therapeutischer Maßnahmen durch die Patientin bzw. ihren gesetzlichen Stellvertreter regelhaft überprüft. Andernfalls kann Übertherapie resultieren. Speziell ausgebildete „communication facilitators“ können bei der Bewältigung der oft komplexen Kommunikationsaufgaben behilflich sein und Übertherapie vermeiden helfen [8].

## Erkennen und Bewerten einer möglichen Überversorgung

Die Verantwortung, bedarfsgerechte und ressourcenbewusste Entscheidungen aufgrund einer wissenschaftlich begründeten und individuell abgestimmten Indikationsstellung zu treffen, obliegt den behandelnden Ärztinnen [20]. Die Arzt-Patienten-Interaktion ist dabei der zentrale Ansatzpunkt, um Überversorgung zu erkennen, zu bewerten und zu vermeiden.

### Bewertung der Indikationsstellung und des Therapieziels unter Berücksichtigung des Patientenwillens

Patientenwille und medizinische Indikation sind Grundlage für die Definition des übergeordneten Therapieziels [48]. Erst das Begreifen des Therapieziels als absolut maßgebend für die Indikationsstellung von Diagnostik und Therapie einerseits und die kontinuierliche Überprüfung seiner Gültigkeit unter Berücksichtigung der Gesamtheit der erhobenen Befunde und des Patientenwillens andererseits lassen Überversorgung erkennen. Die folgenden *5 Leitfragen* können dabei in der Praxis (beispielsweise im Rahmen der täglichen intensivmedizinischen Visite) helfen.

#### Leitfragen

Entscheidungsgrundlage

1. Wie lautet das übergeordnete Therapieziel?
2. Haben sich seit der letzten Reevaluation Befunde oder Änderungen/Erkenntnisse hinsichtlich des Patientenwillens ergeben, vor deren Hintergrund das Therapieziel kritisch überdacht und ggf. neu definiert werden muss?

Identifikation von Überversorgung auf der Indikationsebene

3. Hat jede unserer geplanten diagnostischen Maßnahmen eine Konsequenz, mit der wir dem Erreichen des Therapieziels näherkommen oder aus der wir ein neues Therapieziel ableiten würden?
4. Ist jede einzelne unserer geplanten oder laufenden therapeutischen Maßnahmen geeignet, notwendig und angemessen, um das Therapieziel zu erreichen?

Identifikation von Überversorgung auf Ebene des Patientenwillens

5. Entsprechen unsere geplanten und laufenden diagnostischen und therapeutischen Maßnahmen (weiterhin) dem Patientenwillen?

**Figure 2:**
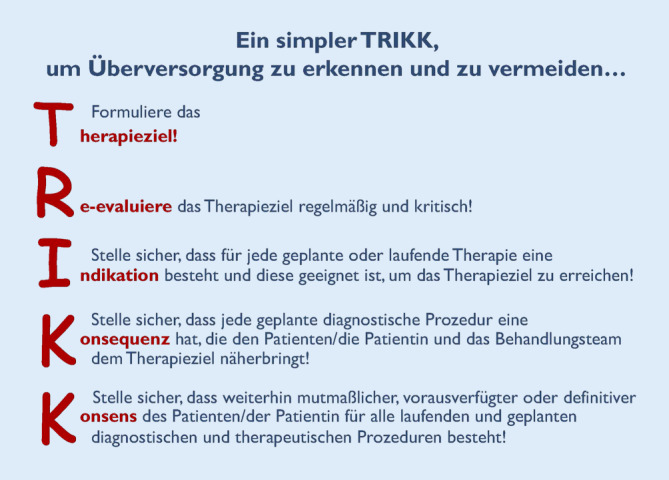

Das *Akronym TRIKK* kann helfen, diese Fragen während der Visite oder im kollegialen Gespräch leicht zu rekapitulieren und strukturiert abzuarbeiten (Abb. 2).

### Reflektiertes Entscheiden als Resultat ständiger Fortbildung und eines kritischen kollegialen Austauschs

Entscheidende Voraussetzung zur Beurteilung, ob diagnostische und therapeutische Maßnahmen geeignet sind, das Therapieziel zu erreichen, ist die Kenntnis der verfügbaren Evidenz. Neben systematisch aufgearbeiteten Informationen, Entscheidungshilfen und ggf. Leitlinien (beispielsweise im Rahmen der Initiativen „Gemeinsam klug entscheiden“ der Arbeitsgemeinschaft Wissenschaftlich Medizinischer Fachgesellschaften oder „Klug entscheiden“ der Deutschen Gesellschaft für Innere Medizin; [55, 62, 67]) spielen hierbei Zweitmeinungsverfahren eine entscheidende Rolle. Um Diagnostik und Therapie bereits so früh wie möglich und so gezielt wie möglich zu betreiben, können die beiden folgenden *Leitfragen* helfen.

#### Leitfragen

1. Verstehen wir die aktuellen Erkrankungsbilder und Verdachtsdiagnosen?
2. Falls nein, haben wir rechtzeitig weitergehende Expertise eingeholt?

## Allgemeine Forderungen zur Vermeidung einer Überversorgung

Aufgrund der komplexen Natur von Überversorgung in der Intensivmedizin und ihren vielgestaltigen Einflussfaktoren und Ursachen [43] lässt sie sich nur durch eine multidimensionale Herangehensweise reduzieren und vermeiden. Das erfordert also gleichzeitig Maßnahmenbündel auf den verschiedenen Ebenen des Gesundheitssystems – also auf Mikro‑, Meso- und Makroebene. Die einzelne Intensivmedizinerin mag zwar auf der normativen Ebene selbst nicht viel verändern können, ihr Einfluss auf die Leitungsebene mag eingeschränkt sein. Aber ihre professionsethische Verpflichtung, auf der Ebene des Behandlungsteams gegen Überversorgung vorzugehen und sie gegenüber den Verantwortlichen auf der Leitungsebene zu beanstanden, bleibt ausdrücklich bestehen [36].

### Forderungen und Empfehlungen auf der Ebene der Behandlungsteams (Mikroebene)

#### Therapieziele und -maßnahmen regelmäßig (re)evaluieren

Ärztinnen sind in der Verantwortung, wissenschaftlich begründete und individuell abgestimmte Indikationsstellungen zu treffen, die sowohl angemessen als auch ressourcenbewusst sind [54]. Dazu bedarf es der gemeinsamen Bewertung eines medizinisch sinnvollen Behandlungsumfangs im Team und dann der Entscheidungsfindung von Patientin und Ärztin und einer kontinuierlichen und vertrauensvollen Betreuung von Patientin und Angehörigen [30, 41]. Die Frage nach der Indikation und dem Therapieziel in Übereinstimmung mit dem Patientenwillen muss nicht nur initial, sondern auch im weiteren Verlauf regelmäßig, z. B. im Rahmen der Visite oder der strukturierten Patienten- und Angehörigenkommunikation, reflektiert werden, um Überversorgung in der laufenden Therapie zu erkennen. Die getroffenen Therapieentscheidungen müssen dann für alle Beteiligten transparent und nachvollziehbar kommuniziert und dokumentiert werden [50].

#### Vorhandene Unterstützungsangebote für das Behandlungsteam, Patienten und Angehörige nutzen

Verschiedene Formen klinischer Ethikberatung und Integration weiterer Spezialisten aus den Bereichen Palliativmedizin, klinische Psychologie/Psychosomatik, Seelsorge, Sozialdienst sind zunehmend verbreitet und sollen die Betroffenen dabei unterstützen, eine sorgfältig abgewogene und gut begründbare Entscheidung zu treffen und dabei entstehende Konflikte zu lösen [2, 9–11]. Insbesondere das Modell der proaktiven Visitenbegleitung durch klinische Ethiker oder Palliativmediziner – unter Wahrung von Schweigepflicht und Datenschutz – kann hilfreich sein, um potenzielle Überversorgung zu erkennen und gemeinsam ein neues Therapiekonzept zu erarbeiten [69–71].

#### Patientenautonomie in der Arzt-Patienten-Beziehung fördern

Intensivmedizinerinnen können durch die angemessene Ausgestaltung der Kommunikation mit den Patienten bzw. ihren gesetzlichen Stellvertretern und den Angehörigen deren Selbstbestimmung und Entscheidungsfähigkeit fördern, insbesondere auch im Rahmen der strukturierten Kommunikation. Dadurch können sie vielfältigen Ängsten und Unsicherheiten als bedeutsamen Einflussfaktoren für das Entstehen von Übertherapie entgegenwirken [21]. Für juristische Stellvertreter (Bevollmächtigte und Betreuer) mag es mitunter schwierig sein, eigene Interessen im Hinblick auf den Patientenwillen zu erkennen und zurückzustellen [49]. Gerade Familienangehörige befinden sich bei der Entscheidungsfindung nicht selten in einer akuten Belastungssituation, bei der sie Unterstützung durch das Behandlungsteam erhalten sollten – sonst resultiert allzu oft eine unreflektierte Fortführung der nicht mehr indizierten lebenserhaltenden intensivmedizinischen Maßnahmen.

### Forderungen und Empfehlungen auf Leitungsebene (Mesoebene)

#### Patientenzentrierte Unternehmenskultur im Krankenhaus leben

Managemententscheidungen im Krankenhaus müssen sich vorrangig an qualitativ hochwertiger Patientenversorgung im Sinne des Versorgungsauftrags („ressourcenbewusste Daseinsfürsorge“; [54]) orientieren statt an betriebswirtschaftlichen Zielgrößen (z. B. Erlössteigerung). Dazu ist eine gemeinsame und unabhängige Krankenhausführung – ärztliche Direktion, Pflegedirektion, kaufmännische Leitung – erforderlich. Gemeinsam sollten Strategien entwickelt werden, die innerhalb der Organisation dazu beitragen können, Überversorgung aufzudecken (z. B. durch Implementierung von „Checklisten“) und zu vermeiden. Insbesondere müssen die Personalausstattung und die Arbeitsbedingungen den Behandlungsteams eine evidenzbasierte, patientenzentrierte Versorgung ermöglichen. Kennziffern der Unternehmenskultur werden bereits durch regelmäßige Befragungen von Mitarbeiterinnen und Patientinnen/Angehörigen erhoben. Diese Instrumente sollten zukünftig auch durch Fragen zur Wahrnehmung einer Übertherapie ergänzt werden [22].

#### Strukturen zur Stärkung der interdisziplinären und interprofessionellen Kommunikation und Kooperation schaffen

Aus organisationsethischer Perspektive ist der Krankenhausträger in der Verantwortung, strukturelle Voraussetzungen zu schaffen, die die interdisziplinäre und interprofessionelle Kommunikation und Kooperation stärken [40, 76]. Dies umfasst unter anderem die Bereithaltung von Unterstützungsstrukturen wie klinische Ethikkomitees und Palliativteams [1, 2]. Eine Ressourcenumverteilung zugunsten einer angemessenen personellen Ausstattung sowie einer nachhaltigen Qualifikation und Wertschätzung des Personals kann dem bestehenden problematischen Personalmangel gegensteuern und Übertherapie begrenzen helfen [52]. Ebenso können durch die Einführung von strukturierten – beispielsweise checklistenbasierten – Prozessen für Aufnahmen, Übergaben und Verlegungen sowie für interdisziplinäre und transsektorale Absprachen das Risiko von Komplikationen und die Gefahr von inadäquaten Maßnahmen minimiert werden [24].

#### Interdisziplinäre und interprofessionelle Zusammenarbeit in Aus‑, Fort- und Weiterbildung stärken

Eine adäquate berufs- und fachgruppenübergreifende Zusammenarbeit gilt als Voraussetzung für eine qualitativ hochwertige Patientenversorgung. Die Forderung zahlreicher Expertengremien (unter anderem des Wissenschaftsrats [79] und der Gesellschaft für Medizinische Ausbildung [77]) besteht deshalb in einer frühzeitigen und stetig wiederkehrenden interprofessionellen Aus‑, Fort- und Weiterbildung, die sich als probates Mittel für die Verbesserung der Ergebnisqualität erwiesen hat [60]. Multiprofessionelle Fort- und Weiterbildungen kommen in der Intensivmedizin im Rahmen der Einübung von Notfallszenarien bereits vielfach zum Einsatz [57, 68]. Jenseits dieses gemeinsamen Simulationstrainings sind inter- oder multiprofessionelle Formate jedoch nach wie vor kein fester und obligatorischer Bestandteil der Fachweiterbildung bzw. Facharztausbildung in der Intensivmedizin. Die curriculare Verankerung von Themen der Über‑, Unter- und Fehlversorgung ist daher weiter voranzutreiben und die Umsetzung konsequent einzufordern.

### Forderungen und Empfehlungen auf gesellschaftlicher, gesundheitspolitischer und gesetzgebender Ebene (Makroebene)

#### Fehlanreize im Krankenhausfinanzierungssystem minimieren

Der Wettbewerb der Krankenhäuser trägt Merkmale ruinöser Konkurrenz, führt zu Defiziten in der Behandlungsqualität und zu Effizienzverlusten [35]. Das Ziel des Wettbewerbs war ursprünglich die Unterbindung von Ressourcenverschwendung und die Schließung von Klinikbetten. Die Folge des Wettbewerbs ist aber eine betriebswirtschaftlich begründete Ausweitung von Leistungen unter Inkaufnahme einer illegitimen und ökonomisch sinnwidrigen Überversorgung. Grundlegende Veränderungen der Krankenhausstrukturen und der Finanzierung sind bislang ausgeblieben [13, 35], was als fehlender politischer Handlungswillen bewertet werden muss. Einer Debatte um eine zentrale Krankenhausbedarfsplanung und eine Priorisierung in der Gesundheitsversorgung, die in anderen Ländern Europas offen geführt wird, hat sich die deutsche Gesundheitspolitik bislang konsequent entzogen.

Die Anwendung des fallpauschalbasierten Vergütungssystems bedarf – nicht nur aus Sicht der Intensivmedizin und -pflege – dringend einer Reformierung. Anreize zur Fallzahlsteigerung und Leistungsausweitung aus ökonomischen Gründen sollten nicht weiterhin gesetzt werden. Konservative und interventionelle/operative Medizin sollten gleich bewertet werden [54, 63]. Ziel muss es sein, den ökonomischen Druck aus der Beatmungstherapie und anderen invasiven intensivmedizinischen Prozeduren zu nehmen, um bei der Entscheidung für oder gegen eine bestimmte Therapiemaßnahme die Abwägung des Patientenwillens mit der Indikation in den Mittelpunkt stellen zu können [63]. Fehlanreize für Übertherapie könnten so gezielt minimiert werden. Die Bedeutung und Wertschätzung einer kritischen ethischen Reflexion im Behandlungsteam sollte sich auch darin ausdrücken, dass sich definierte Formen von Ethikberatung im Vergütungssystem abbilden.

#### Gesellschaftlichen Diskurs zur Überversorgung anstoßen

Die Vermeidung einer Überversorgung erfordert auch ein Umdenken innerhalb der Gesellschaft, die weithin von einer ausgeprägten Vorstellung der Omnipotenz der Intensivmedizin gekennzeichnet ist und daraus umfassende (Versorgungs‑)Ansprüche ableitet. Es gilt deshalb, auch in der Allgemeinbevölkerung einen umfassenden Diskurs über die Möglichkeiten und Grenzen von Intensivmedizin anzuregen und qualifiziert zu begleiten. Dazu ist auch ein Diskurs über unrealistische Gesundheitsideale und unreflektierte Optimierungsvorstellungen erforderlich. Anreize sollten eher der Gesunderhaltung und dem gelingenden Umgang mit Alter(n), Krankheit und Beeinträchtigungen dienen als einer nachträglichen Reparatur von Organen und Körperfunktionen.

#### Umfassende Vorsorgeplanung einführen, ausweiten und evaluieren

Vorausverfügte Willensäußerungen (schriftlich oder mündlich) und die Benennung von Vorsorgebevollmächtigten können jedem einzelnen (potenziellen) Patienten helfen, ungewünschte intensivmedizinische Maßnahmen zu verhindern. Schwierigkeiten mit konventionellen Patientenverfügungen und deren Anwendung auf der Intensivstation sind durch zahlreiche empirische Untersuchungen aufgedeckt worden [32]. Das Konzept des Advance Care Planning (ACP), das die vorhandenen Schwachstellen adressiert [23, 83], ist jedoch bislang nicht flächendeckend und auf allen relevanten Ebenen realisiert. Auch die Voraussetzungen für die Kostenübernahme für ACP sind gesetzlich nur für ausgewählte Bereiche geklärt (vgl. § 132g SGB V). Vor allem für Intensivmedizinerinnen könnte aus einer breiteren, sektorenübergreifenden Einführung mehr Handlungssicherheit im Einzelfall resultieren, die dazu beitragen kann, Übertherapie zu vermeiden und eine Therapie durchzuführen, die an den tatsächlichen individuellen Bedürfnissen der Patientin orientiert ist.

## Zusammenfassung

Überversorgung, also eine unangemessen umfangreiche Therapie, ist ein ernstzunehmendes negatives Phänomen auch auf Intensivstationen im deutschen Gesundheitssystem. Sie kann einerseits Patienten, Angehörigen und den Behandlungsteams schaden und führt andererseits durch Ressourcenverschwendung zum Schaden für die Allgemeinheit.

Überversorgung ist einem Konglomerat mehrerer Ursachen geschuldet. Wesentlicher Faktor ist die Verquickung von unbedachter, unangemessener oder fehlerhafter Indikationsstellung mit falschen Vorstellungen und Erwartungen in Bezug auf Nutzen und Risiken intensivmedizinischer Behandlungsmaßnahmen, insbesondere auf Seiten der Patientinnen oder ihrer gesetzlichen Stellvertreter.

Auf normativer Ebene sind gesellschafts- und gesundheitspolitische Fehlentwicklungen zu korrigieren, die allerdings auf der Ebene der Behandlungsteams kaum zu beeinflussen sind. Umso mehr bleibt es berufsethische ärztliche Verpflichtung, ausgehend vom medizinisch sinnvoll Möglichen im Behandlungsteam einen angemessenen Behandlungsumfang für jede Patientin individuell festzulegen, der deren Willen bestmöglich entspricht. Im vorliegenden Positionspapier sind im Akronym TRIKK 5 Leitfragen für intensivmedizinische Behandlungsteams zusammengefasst, die dabei helfen können, Überversorgung zu erkennen und zu reduzieren – bestenfalls sogar zu vermeiden.

Die dargestellten Argumente und Forderungen haben auch Bestand während außergewöhnlicher Belastungen wie der aktuellen Pandemie oder zukünftiger Katastrophenfälle. Notfallsituationen setzen Grundregeln für medizinische Entscheidungen nicht außer Kraft. Die COVID-19-Pandemie hat nachdrücklich gezeigt, dass es geboten sein kann, für besondere Situationen eine Notfallreserve bereitzuhalten und die dafür notwendige Finanzierung durchgängig zu sichern. Dabei müssen allerdings räumliche, apparative, logistische und insbesondere personelle Ressourcen bedacht werden. Zugleich ist darauf zu achten, dass eine solcherart vorgehaltene Reserve nicht eine Angebotsausweitung begünstigt, die wiederum einer Überversorgung Vorschub leisten kann.

## References

[1] Adler K, Schlieper D, Kindgen-Milles D (2017). Integration der Palliativmedizin in die Intensivmedizin : Systematische Ubersichtsarbeit. Anaesthesist.

[2] Au SS, Couillard P, Roze des Ordons A (2018). Outcomes of ethics consultations in adult ICus: a systematic review and meta-analysis. Crit Care Med.

[3] Beinersdorf A (2007). Der Informationsverlust bei der Patientenverlegung. intensiv.

[4] Benoit DD, Jensen HI, Malmgren J (2018). Outcome in patients perceived as receiving excessive care across different ethical climates: a prospective study in 68 intensive care units in Europe and the USA. Intensive Care Med.

[5] Bion J, Antonelli M, Blanch L (2018). White paper: statement on conflicts of interest. Intensive Care Med.

[6] Boothroyd LJ, Spaziano M, Guertin JR (2013). Transcatheter aortic valve implantation: recommendations for practice based on a multidisciplinary review including cost-effectiveness and ethical and organizational issues. Can J Cardiol.

[7] Consortium WHOST, Pan H, Peto R (2020). Repurposed antiviral drugs for Covid-19—interim WHO solidarity trial results. N Engl J Med.

[8] Curtis JR, Treece PD, Nielsen EL (2016). Randomized trial of communication facilitators to reduce family distress and intensity of end-of-life care. Am J Respir Crit Care Med.

[9] Deffner T, Michels G, Nojack A (2020). Psychologische Versorgung auf der Intensivstation : Tatigkeitsbereiche, Aufgaben, Anforderungen und Ausstattung. Med Klin Intensivmed Notfmed.

[10] Deffner T, Rosendahl J, Niecke A (2020). Psychotraumatologische Aspekte in der Intensivmedizin. Med Klin Intensivmed Notfmed.

[11] Deffner T, Schwarzkopf D, Waydhas C (2019). Psychologische Versorgung auf deutschen Intensivstationen : Ergebnisse einer Umfrage unter den Mitgliedern der Deutschen Interdisziplinaren Vereinigung fur Intensiv- und Notfallmedizin. Med Klin Intensivmed Notfmed.

[12] Deutscher Ethikrat (2014) Vom Krankenhaus zum kranken Haus? Klinikalltag zwischen ethischem Anspruch und Kostendruck. https://www.ethikrat.org/weitere-veranstaltungen/vom-krankenhaus-zum-kranken-haus-klinikalltag-zwischen-ethischem-anspruch-und-kostendruck/. Zugegriffen: 23. Aug. 2020

[13] Deutscher Ethikrat (2011) Nutzen und Kosten im Gesundheitswesen – Zur normativen Funktion ihrer Bewertung. https://www.ethikrat.org/fileadmin/Publikationen/Stellungnahmen/deutsch/DER_StnAllo-Aufl2_Online.pdf. Zugegriffen: 23. Aug. 2020

[14] Druml W, Druml C (2019). Ubertherapie in der Intensivmedizin. Med Klin Intensivmed Notfmed.

[15] Duttge G, Er D, Fischer ES, Steinfath H, Wiesemann C (2016). Vertrauen durch Recht?. Autonomie und Vertrauen – Schlüsselbegriffe der modernen Medizin.

[16] Eddy DM (1984). Variations in physician practice: the role of uncertainty. Health Aff.

[17] Elshaug AG, Rosenthal MB, Lavis JN (2017). Levers for addressing medical underuse and overuse: achieving high-value health care. Lancet.

[18] Fleischmann-Struzek C, Mikolajetz A, Reinhart K (2019). Hospitalisierung und Intensivtherapie am Lebensende. Dtsch Arztebl Int.

[19] Grignoli N, Di Bernardo V, Malacrida R (2018). New perspectives on substituted relational autonomy for shared decision-making in critical care. Crit Care.

[20] Grote Westrick M, Volbracht E (2020). Überversorgung – Ausmaß, Ursachen und Gegenmaßnahmen. GGW.

[21] Hambrock U (2019) Erfahrungen mit Überversorgung: Qualitativ-psychologische Studie mit Patienten und Ärzten der Bertelsmann Stiftung. https://www.bertelsmann-stiftung.de/de/publikationen/publikation/did/erfahrungen-mit-ueberversorgung/. Zugegriffen: 7. Dez. 2020

[22] Hartog CS, Hoffmann F, Mikolajetz A (2018). Ubertherapie und emotionale Erschopfung in der „end-of-life care“ : Ergebnisse einer Mitarbeiterumfrage auf der Intensivstation. Anaesthesist.

[23] Hartog CS, Spies CD, Michl S (2020). Advance Care Planning in Zeiten der Corona-Pandemie – eine Chance fur die Patientenautonomie in der Akutsituation. Med Klin Intensivmed Notfmed.

[24] Haynes AB, Weiser TG, Berry WR (2009). A surgical safety checklist to reduce morbidity and mortality in a global population. N Engl J Med.

[25] Hofmann B (2019). Expanding disease and undermining the ethos of medicine. Eur J Epidemiol.

[26] Hofmann B (2018). Looking for trouble? Diagnostics expanding disease and producing patients. J Eval Clin Pract.

[27] Ilkilic I (2008). Kulturelle Aspekte bei ethischen Entscheidungen am Lebensende und interkulturelle Kompetenz. Bundesgesundheitsblatt Gesundheitsforschung Gesundheitsschutz.

[28] Klemperer D (2008). Gefahr für das ärztliche Urteilsvermögen ocesses. Dtsch Arztebl Int.

[29] Kohn R, Rubenfeld GD, Levy MM (2011). Rule of rescue or the good of the many? An analysis of physicians’ and nurses’ preferences for allocating ICU beds. Intensive Care Med.

[30] Kon AA, Davidson JE, Morrison W (2016). Shared decision making in ICus: an American College of Critical Care Medicine and American Thoracic Society policy statement. Crit Care Med.

[31] Kopp R, Wildenauer R, Marx G (2020). Umsichtig und vernünftig handeln auf der operativen Intensivstation. Anasth Intensivmed.

[32] Langer S, Knorr J-U, Berg A (2013). Umgang mit Patientenverfügungen: Probleme durch pauschale Formulierungen. Dtsch Arztebl Int.

[33] Leopoldina Nationale Akademie der Wissenschaften (2016) Zum Verhältnis von Medizin und Ökonomie im deutschen Gesundheitssystem: 8 Thesen zur Weiterentwicklung zum Wohle der Patienten und der Gesellschaft. https://www.leopoldina.org/publikationen/detailansicht/publication/zum-verhaeltnis-von-medizin-und-oekonomie-im-deutschen-gesundheitssystem-2016/. Zugegriffen: 23. Aug. 2020

[34] Long AC, Brumback LC, Curtis JR (2019). Agreement with consensus statements on end-of-life care: a description of variability at the level of the provider, hospital, and country. Crit Care Med.

[35] Loos S, Albrecht M, Zich K (2019) Zukunftsfähige Krankenhausversorgung: Simulation und Analyse einer Neustrukturierung der Krankenhausversorgung am Beispiel einer Versorgungsregion in Nordrhein-Westfalen. https://www.bertelsmann-stiftung.de/de/publikationen/publikation/did/zukunftsfaehige-krankenhausversorgung. Zugegriffen: 23. Aug. 2020

[36] Maio G, Katzenmeier C, Bergdolt K (2009). Dienst am Menschen oder Kunden-Dienst? Ethische Grundreflexionen zur sich wandelnden ärztlichen Identität. Das Bild des Arztes im 21. Jahrhundert.

[37] Marshall AP, Tobiano G, Murphy N (2019). Handover from operating theatre to the intensive care unit: A quality improvement study. Aust Crit Care.

[38] McGauran N, Wieseler B, Kreis J (2010). Reporting bias in medical research—a narrative review. Trials.

[39] Metaxa V, Ely EW, Michalsen A, Sadovnikoff N (2020). Cultural diversity. Compelling ethical challenges in critical care and emergency medicine.

[40] Michalsen A, Hillert A, Kluge S, Marx C, Janssens U, Zacharowski K (2018). Stressreduktion und Burn-out-Prophylaxe. Management in der Intensivmedizin.

[41] Michalsen A, Long AC, DeKeyser Ganz F (2019). Interprofessional shared decision-making in the ICU: a systematic review and recommendations from an expert panel. Crit Care Med.

[42] Moorhead SA, Hazlett DE, Harrison L (2013). A new dimension of health care: systematic review of the uses, benefits, and limitations of social media for health communication. J Med Internet Res.

[43] Morgan DJ, Leppin AL, Smith CD (2017). A practical framework for understanding and reducing medical overuse: conceptualizing overuse through the patient-clinician interaction. J Hosp Med.

[44] Moss M, Good VS, Gozal D (2016). An official critical care societies collaborative statement: burnout syndrome in critical care health care professionals: a call for action. Am J Crit Care.

[45] Müller R, Ganten D, Larisch J (2014). Public Health: Gesundheit ist mehr als Medizin. Dtsch Arztebl Int.

[46] Mulley AG, Trimble C, Elwyn G (2012). Stop the silent misdiagnosis: patients’ preferences matter. BMJ.

[47] Neitzke G (2019). Ermittlung des Patientenwillens. Anasthesiol Intensivmed Notfallmed Schmerzther.

[48] Neitzke G (2014). Indikation: fachliche und ethische Basis ärztlichen Handelns. Med Klin Intensivmed Notfmed.

[49] Neitzke G (2019). Juristische Stellvertreter in der Medizin: Bevollmächtigte und Betreuer. Anasthesiol Intensivmed Notfallmed Schmerzther.

[50] Neitzke G, Boll B, Burchardi H (2017). Dokumentation Therapiebegrenzung – Empfehlung der Sektion Ethik der Deutschen Interdisziplinäre Vereinigung für Intensiv und Notfallmedizin (DIVI) unter Mitarbeit der Sektion Ethik der Deutschen Gesellschaft für Internistische Intensivmedizin und Notfallmedizin (DGIIN). Med Klin Intensivmed Notfmed.

[51] Neitzke G, Burchardi H, Duttge G (2016). Grenzen der Sinnhaftigkeit von Intensivmedizin. Med Klin Intensivmed Notfmed.

[52] Neuraz A, Guerin C, Payet C (2015). Patient mortality is associated with staff resources and workload in the ICU: a multicenter observational study. Crit Care Med.

[53] Noordman J, van Vliet L, Kaunang M (2019). Towards appropriate information provision for and decision-making with patients with limited health literacy in hospital-based palliative care in Western countries: a scoping review into available communication strategies and tools for healthcare providers. BMC Palliat Care.

[54] Nothacker M, Busse R, Elsner P (2019). Medizin und Okonomie: Massnahmen fur eine wissenschaftlich begrundete, patientenzentrierte und ressourcenbewusste Versorgung. Ein Strategiepapier der Arbeitsgemeinschaft der Wissenschaftlichen Medizinischen Fachgesellschaften (AWMF). Dtsch Med Wochenschr.

[55] Nothacker M, Kreienberg R, Kopp IB (2017). „Gemeinsam Klug Entscheiden“ – eine Initiative der AWMF und ihrer Fachgesellschaften: Mission, Methodik und Anwendung. Z Evid Fortbild Qual Gesundhwes.

[56] OECD (2017). Tackling wasteful spending on health.

[57] Partecke M, Balzer C, Finkenzeller I (2016). Interprofessionelles Lernen an der Universitätsmedizin Greifswald – Didaktische Konzeption und praktische Etablierung eines notfallmedizinischen Teamtrainings von Medizinstudierenden und Auszubildenden der Gesundheits- und Krankenpflege. GMS J Med Educ.

[58] Pfister H-R, Jungermann H, Fischer K (2016). Die Psychologie der Entscheidung.

[59] Ranieri VM, Thompson BT, Barie PS (2012). Drotrecogin alfa (activated) in adults with septic shock. N Engl J Med.

[60] Reeves S, Perrier L, Goldman J (2013). Interprofessional education: effects on professional practice and healthcare outcomes. Cochrane Database Syst Rev.

[61] Riessen R, Hermes C, Bodmann KF (2018). Vergutung intensivmedizinischer Leistungen im DRG-System : Aktuelle Probleme und Losungsvorschlage. Med Klin Intensivmed Notfmed.

[62] Riessen R, Kluge S, Janssens U (2017). Klug entscheiden – Empfehlungen in der internistischen Intensivmedizin. Internist.

[63] Riessen R, Markewitz A, Grigoleit M (2020). Diskussionspapier fur eine Reform der Krankenhausfinanzierung in Deutschland aus der Perspektive der Intensivmedizin. Med Klin Intensivmed Notfmed.

[64] Robertsen A, Jöbges S, Sadovnikoff N, Michalsen A, Sadovnikoff N (2020). Consent, advance directives, and decision by proxies. Compelling ethical challenges in critical care and emergency medicine.

[65] Sachverständigenrat zur Begutachtung der Entwicklung im Gesundheitswesen (SVR) (2018) Bedarfsgerechte Steuerung der Gesundheitsversorgung. https://www.svr-gesundheit.de/index.php?id=606. Zugegriffen: 7. Dez. 2020

[66] Sadoughi F, Nasiri S, Ahmadi H (2018). The impact of health information exchange on healthcare quality and cost-effectiveness: a systematic literature review. Comput Methods Programs Biomed.

[67] Schaefer C, Klemperer D (2020). Mit Leitlinien, Shared Decision Making und Choosing Wisely gegen Über‑, Unter- und Fehlversorgung?. GGW.

[68] Schindele D, Muller-Wolff T, McDonough JP (2020). Klinische Handlungskompetenzen gemeinsam verbessern – interprofessionelles Lernen in der Intensivmedizin. Med Klin Intensivmed Notfmed.

[69] Schneiderman LJ (2006). Effect of ethics consultations in the intensive care unit. Crit Care Med.

[70] Schneiderman LJ (2005). Ethics consultation in the intensive care unit. Curr. Opin. Crit Care.

[71] Schneiderman LJ, Gilmer T, Teetzel HD (2000). Impact of ethics consultations in the intensive care setting: a randomized, controlled trial. Crit Care Med.

[72] Schreyögg J, Milstein R (2020) Bedarfsgerechte Gestaltung der Krankenhausvergütung – Reformvorschläge unter der Berücksichtigung von Ansätzen anderer Staaten. https://www.hche.uni-hamburg.de/search.html?q=Bedarfsgerechte+Gestaltung. Zugegriffen: 7. Dez. 2020

[73] Schweda M, Schicktanz S, Raz A (2017). Beyond cultural stereotyping: views on end-of-life decision making among religious and secular persons in the USA, Germany, and Israel. BMC Med Ethics.

[74] Sprung CL, Jennerich AL, Joynt GM et al (2020) The influence of geography, religion, religiosity and institutional factors on worldwide end-of-life care for the critically ill: the WELPICUS study. J Palliat Care 10.1177/08258597211002308 33818159

[75] Stüwe H (2015). Private Klinikträger: Die Großen erzielen gute Gewinne. Dtsch Arztebl Int.

[76] Van den Bulcke B, Metaxa V, Reyners AK (2020). Ethical climate and intention to leave among critical care clinicians: an observational study in 68 intensive care units across Europe and the United States. Intensive Care Med.

[77] Walkenhorst U, Mahler C, Aistleithner R (2015). Positionspapier GMA-Ausschuss – Interprofessionelle Ausbildung in den Gesundheitsberufen. GMS Z Med Ausbild.

[78] Wieland W (1986). Strukturwandel der Medizin und ärztliche Ethik. Philosophische Überlegungen zu Grundfragen einer praktischen Wissenschaft.

[79] Wissenschaftsrat (2014) Empfehlungen zur Weiterentwicklung des Medizinstudiums in Deutschland auf Grundlage einer Bestandsaufnahme der humanmedizinischen Modellstudiengänge. https://www.wissenschaftsrat.de/download/archiv/4017-14.pdf. Zugegriffen: 7. Dez. 2020

[80] Wohlmannstetter M (2019). Überpflege – gibt es das auch?. Med Klin Intensivmed Notfmed.

[81] Wray CM, Loo LK (2015). The diagnosis, prognosis, and treatment of medical uncertainty. J Grad Med Educ.

[82] Zander B, Dobler L, Baumler M (2014). Implizite Rationierung von Pflegeleistungen in deutschen Akutkrankenhausern – Ergebnisse der internationalen Pflegestudie RN4Cast. Gesundheitswesen.

[83] Zentrale Ethikkommission (2020). Stellungnahme der Zentralen Kommission zur Wahrung ethischer Grundsätze in der Medizin und ihren Grenzgebieten (Zentrale Ethikkommission) bei der Bundesärztekammer: „Außerklinische Ethikberatung“. Dtsch Arztebl Int.

